# Early-Onset Glaucoma in *egl1* Mice Homozygous for *Pitx2* Mutation

**DOI:** 10.3390/biomedicines10030516

**Published:** 2022-02-22

**Authors:** Bindu Kodati, Shawn A. Merchant, J. Cameron Millar, Yang Liu

**Affiliations:** 1 North Texas Eye Research Institute, Department of Pharmacology and Neuroscience, University of North Texas Health Science Center, Fort Worth, TX 76107, USA; bindu.kodati@unthsc.edu (B.K.); cameron.millar@unthsc.edu (J.C.M.); 2 Department of Psychology and Neuroscience, Baylor University, Waco, TX 76798, USA; shawn_merchant1@baylor.edu

**Keywords:** glaucoma, *Pitx2*, mouse model

## Abstract

Mutations in *PITX2* cause Axenfeld–Rieger syndrome, with congenital glaucoma as an ocular feature. The *egl1* mouse strain carries a chemically induced *Pitx2* mutation and develops early-onset glaucoma. In this study, we characterized the glaucomatous features in *egl1* mice. The eyes of *egl1* and C57BL/6J control mice were assessed by slit lamp examination, total aqueous humor outflow facility, intraocular pressure (IOP) measurement, pattern electroretinography (PERG) recording, and histologic and immunohistochemistry assessment beginning at 3 weeks and up to 12 months of age. The *egl1* mice developed elevated IOP as early as 4 weeks old. The IOP elevation was variable and asymmetric within and between the animals. The aqueous humor outflow facility was significantly reduced in 12-month-old animals. PERG detected a decreased response at 2 weeks after the development of IOP elevation. Retinal ganglion cell (RGC) loss was detected after 8 weeks of IOP elevation. Slit lamp and histologic evaluation revealed corneal opacity, iridocorneal adhesions (anterior synechiae), and ciliary body atrophy in *egl1* mice. Immunohistochemistry assessment demonstrated glial cell activation and RGC axonal injury in response to IOP elevation. These results show that the eyes of *egl1* mice exhibit anterior segment dysgenesis and early-onset glaucoma. The *egl1* mouse strain may represent a useful model for the study of congenital glaucoma.

## 1. Introduction

Glaucoma is a multifactorial optic neuropathy characterized by retinal ganglion cell (RGC) loss, which accompanies optic nerve axonal injury. The disease leads to irreversible blindness if left untreated. Elevated intraocular pressure (IOP) is a major risk factor for glaucoma development and progression [1]. Congenital glaucoma, a major cause of childhood blindness, is a severe form of glaucoma that occurs in children from birth to the age of 3 years (infantile glaucoma) or after 3 years of age (juvenile glaucoma). According to the American Academy of Ophthalmology, congenital glaucoma is diagnosed only in 25% of babies who manifest the condition at birth and may occur in as many as 1 in 10,000 live births [2,3]. The increase in IOP in congenital glaucoma is due to developmental abnormalities in the anterior chamber angle, including trabeculodysgenesis, which leads to increased aqueous humor outflow resistance and thus elevated IOP [4,5]. Despite extensive research on congenital glaucoma, the molecular and cellular pathological events that occur are still unclear.

Genetic studies have been conducted to identify genes associated with congenital glaucoma. Research has shown that mutations in certain genes, such as *PITX2*, *FOXC1*, *PAX6,* and *CYP1B1*, are associated with congenital glaucoma [6,7,8,9,10,11]. Among these genes, extensive work has been carried out to identify the functions of *PITX2. PITX2* is involved in the Nodal/Sonic Hedgehog pathway, which determines the polarity and asymmetrical expression during the early development of the mesoderm derived organs as well as the eye, tooth, and umbilicus [12,13,14]. *PITX2* produces three isoforms (*PITX2a, PITX2b*, and *PITX2c*), which consist of similar bicoid-like homeodomain 2 (HD) transcription factors that play an important role in fetal and embryonic development. Missense mutations, or heterozygous defects of the *PITX2* gene in the HD region, cause DNA binding impairment, thereby altering the transactivation of transcription factors, which can lead to ocular deficits by gain or loss of function [15,16,17]. The expression of *PITX2* is higher in the anterior segment than in either the retina or the sclera during the first 9.5 weeks of intrauterine development. *PITX2* expression is further increased in the iridocorneal complex during week 18 [18]. Intragenic deletions and decreased mRNA expression of *PITX2* have been observed in patients with Axenfeld–Rieger syndrome affected by advanced glaucoma [7,19,20]. Using transcription activator-like effector nuclease (TALEN)-mediated genome editing, studies on the zebrafish for the generation of *pitx2*^−/−^ lines have also revealed the importance of *pitx2* in ocular development. Transcriptome studies have identified the molecular changes associated with disease pathology [21]. Similarly, other studies on zebrafish and mouse models have also shown that, as in Axenfeld–Rieger syndrome, *Pitx2* mutations can lead to abnormalities in the development of the anterior segment of the eye as well as protuberant umbilicus, dental hypoplasia, and facial dysmorphism [22,23,24].

Certain mouse strains develop spontaneous glaucoma at a young age. These strains are valuable tools for studying the mechanisms of early-onset glaucoma in humans. A recent study described that mice heterozygous for the *Pitx2* deletion (*Pitx2*^+/−^) modeled the major ocular features of Axenfeld–Rieger syndrome and associated glaucoma [25]. In addition, a mouse strain homozygous for the *Pitx2* mutation named the *egl1* strain has been established in an N-ethyl-N-nitrosourea chemical mutagenesis screening program [26,27]. On the basis of the whole-exome sequencing results of the *egl1* strain, *Pitx2* p.R115L knock-in mice were further generated using CRISPR/Cas9 technology and characterized for a glaucomatous phenotype [26]. The establishment of these models provides a powerful tool to explore mechanisms of glaucoma pathogenesis. Each mouse strain is unique in its development, genotype, and genetic background. The *egl1* mouse strain is commercially available as an early-onset glaucoma mouse model. However, the characterization of this strain has yet to be completed.

In this study, we further characterized the glaucomatous phenotype in the *egl1* mouse strain. We assessed the anterior segment morphology, aqueous humor outflow facility, IOP elevation, and RGC and optic nerve head degeneration. Our study provides insightful information for using the *egl1* mouse strain as an early-onset glaucoma model. 

## 2. Materials and Methods

### 2.1. Animals

C57BL/6J and C57BL/6J-*Pitx2^egl1^*/Boc *(egl1* mutant) mice were obtained from the Jackson Laboratory (Bar Harbor, ME, USA). All animal experiments were performed in accordance with the National Institutes of Health guide for the care and use of laboratory animals and were approved by the Institutional Animal Care and Use Committee of the University of North Texas Health Science Center (IACUC 2020-0023). Mice (both sexes) aged 3 weeks to 12 months were examined. Thirteen C57BL/6J mice (7 females and 6 males) and 60 *egl1* mice (33 females and 27 males) were randomized into the study, and data were obtained from 26 and 116 eyes from C57BL/6J and *egl1* mutant mice, respectively. Exclusion criteria included fluid leaking during aqueous humor outflow facility measurement and central corneal opacity affecting pattern electroretinography recording. Each individual eye was considered the experimental unit in this study. Each experiment includes similar numbers of female and male mice.

All animals were housed in individually ventilated cages (IVCs, polysulfone material with 500 cm^2^ floor space) (Allentown, Allentown, NJ, USA) at a temperature of 21 to 24 °C and humidity of 40–45%. Lights were turned on at 0630 h, and a 12 h light/12 h dark cycle was maintained. Same-sex littermates were housed together at the maximum density of 5 mice per cage. As bedding, a 1/8-inch corn cob (The Andersons, Maumee, OH, USA) was provided. Shredded paper and enrichment rectangles were used as nesting and enrichment (The Andersons). Mice were fed an irradiated mouse diet (5LG4, LabDiet, St. Louis, MO, USA) and provided reverse osmosis filtered drinking water *ad libitum*. All materials, including IVCs, lids, feeders, bedding, nesting, and enrichment, were autoclaved before use. Sentinel mice were negative for at least all Federation of Laboratory Animal Science Associations (FELASA)-relevant murine infectious agents, as monitored by the Department of Laboratory Animal Medicine on campus. 

### 2.2. Intraocular Pressure Measurement

Intraocular pressure (IOP) was measured non-invasively using the TonoLab impact tonometer (Colonial Medical Supply, Franconia, NH, USA) as described previously [28,29]. Briefly, mice were placed in a soft plastic cone (Braintree Scientific, Inc., Braintree, MA, USA) and gently restrained in a plastic mouse restrainer (Colonial Medical Supply, Londonderry, NH, USA). IOP was measured after mice were acclimated. All measurements were performed during the same 3 h time window (1–4 pm) during the lights-on phase of the day; the average of 4–6 measurements was used as the IOP value. IOP was measured twice a week until pressure elevation was detected, and once a week thereafter. Total IOP exposure for each individual eye was determined by the determination of the area under the IOP–time curve (AUC). Six C57BL/6J and twenty-two *egl1* mice were included in this experiment, and IOP was monitored at age 3 through 8 weeks old.

### 2.3. Slit Lamp Examination

Anterior segments of mouse eyes were examined with a slit lamp (SL-D7; Topcon, Tokyo, Japan), and images were taken with a digital camera (D100; Nikon, Tokyo, Japan). Slit lamp examinations were performed on conscious animals. Six C57BL/6J and twenty-two *egl1* mice were examined longitudinally at age 4 weeks to 12 months.

### 2.4. Anterior Segment Histologic Examination

For histologic examination, animals were euthanized by exposure to 10% to 30% cage volume/min carbon dioxide. After death was confirmed, eyes were enucleated and fixed in 10% neutral formalin (Electron Microscopy Sciences, Hatfield, PA, USA) overnight. Eyes were then dehydrated with ethanol and xylene and embedded in paraffin. Sagittal sections (5 µm) were prepared, mounted on glass microscope slides, and stained with hematoxylin and eosin (H&E) for structural evaluation. Three C57BL/6J and six *egl1* mice were examined before and 4 weeks after intraocular pressure elevation.

### 2.5. Immunofluorescent Staining

For immunofluorescent staining, eyes were fixed in 4% paraformaldehyde (Electron Microscopy Sciences) in phosphate-buffered saline (PBS) for 2 h at 4 °C and cryo-preserved after 10%, 20%, and 30% sucrose (Thermo Scientific, Rockford, IL, USA) sequential cryo-protection. Cryosections of mouse eyes were blocked with PBS-based SuperBlock (Thermo Scientific) for 2 h at room temperature (RT) and incubated overnight at 4 °C with primary antibodies against glial fibrillary acidic protein (GFAP, 1:500 dilution; Cell Signaling #3670, Danvers, CA, USA) [30], ionized calcium-binding adaptor 1 (Iba-1, 1:500 dilution, Fujifilm Cellular Dynamics 019-19741, Madison, WI, USA) [31], or neurofilament H (NF-H, 1:1000 dilution, Abcam ab8135, Waltham, MA, USA) [32]. After 3 rinses in PBS, sections were further incubated with Alexa488 or TRITC conjugated secondary antibodies (Life Technologies, Carlsbad, CA, USA) against rabbit (for Iba-1 and NF-H) or mouse (for GFAP) IgGs for 1 h at RT. The sections were rinsed again and mounted in ProLong Gold anti-fade reagent with DAPI (Thermo Scientific). Non-primary control staining was performed using PBS instead of primary antibodies. Images were viewed and captured using a Zeiss LSM 510 META confocal microscope. The fluorescence intensity for Iba-1 and GFAP were analyzed using ImageJ software in a masked manner. Two C57BL/6J and seven *egl1* mice (2–3 mice per time point) were included in this experiment. 

### 2.6. Aqueous Humor (AH) Outflow Facility Measurement

The *egl1* mice at ages 8 weeks and 1 year were used for aqueous humor outflow facility measurement, which was performed using a constant flow infusion method established previously [33,34,35,36]. In brief, mice were anesthetized by an intraperitoneal injection of a cocktail of ketamine/xylazine (100/10 mg/kg, respectively; maintenance: 1/2 × to 1/4 × induction dose). One drop of 0.5% proparacaine HCl was applied for corneal anesthesia (Alcaine, Alcon, Fort Worth, TX, USA). The anterior chamber of each eye was cannulated with a 32-gauge needle attached to tubing connected to a pressure transducer (BLPR2; World Precision Instruments (WPI), Sarasota, FL, USA) and a glass microsyringe (Hamilton Company, Reno, NV, USA) filled with sterile PBS and loaded onto a microdialysis infusion pump (SP101i; WPI). The eyes were infused at a flow rate of 0.1 µL/min initially for approximately 30 min to stabilize the pressure registered by the pressure transducer. On pressure stabilization, 3 pressure readings, spaced 5 min apart, were obtained over the following 10 min period. The flow rate was then increased to 0.2 µL/min, and following 5 min for stabilization, 3 pressure readings were obtained in a similar manner. The process was then repeated at flow rates of 0.3, 0.4, and 0.5 µL/min. Mean stabilized pressure flow rate curves were generated for each eye and fit using simple linear regression. The total aqueous humor outflow facility was calculated as the reciprocal of the slope of each respective curve. All measurements were conducted in a single masked manner. Seven *egl1* mice (3 aged 2 months and 4 aged 12 months) were assessed.

### 2.7. Retinal Ganglion Cell Function Assessment

Pattern electroretinography (PERG) was performed in 6 *egl1* mice using the JORVEC System (Intelligent Hearing Systems, Miami, FL, USA) as described previously [37]. The *egl1* mice with severe corneal opacity that blocked central cornea were excluded from the PERG study. Briefly, mice were anesthetized with intraperitoneal injections of a mixture of ketamine and xylazine (100 and 10 mg/kg, respectively). The PERG responses were recorded from a stainless-steel needle (Grass, West Warwick, RI, USA) placed in the snout subcutaneously. Pattern stimuli consisting of contrast-reversing gratings with a spatial frequency of 0.05 cycles/deg and maximum contrast were displayed on two custom-made tablets. The contrast reversal frequency was 1 Hz. A total of 2232 responses were averaged. The amplitude was measured from the positive peak to the negative trough, and the latency was the time to the peak of the response.

### 2.8. Retinal Ganglion Cell Quantification

Quantification of retinal ganglion cells (RGCs) was performed using immunostained retinal whole mounts [38]. Briefly, mice (5 C57BL/6J and 21 *egl1* mice) were euthanized by exposure to 100% CO_2(g)_. Following the cessation of breathing and heartbeat, eyes were enucleated and fixed in 4% paraformaldehyde (Electron Microscopy Sciences) for 2 h at 4 °C. Retinas were dissected from fixed eyes and blocked with 0.3% Triton X-100 in PBS containing 2% goat serum for 2 h. Retinas were incubated in rabbit polyclonal RBPMS antibody (1:200, diluted in 0.3% Triton X-100 in PBS, GeneTex GTX118619, Irvine, CA, USA) [39] overnight at 4 °C. Following washes in PBS, retinas were further incubated in AlexaFluor596 goat-anti-rabbit (1:1000, diluted in 0.1% Triton X-100 in PBS) overnight at 4 °C. After washes with PBS, the retinas were cut into four quadrants and mounted on glass slides (Fischer Scientific, Pittsburgh, PA, USA). Eight images were taken from the peripheral and mid-peripheral regions in four quadrants of each retina. The number of cells from each image (0.0867 mm^2^ retina area) was counted using Adobe Photoshop software V22.2 (Adobe Systems, Inc., San Jose, CA, USA). The average of all counts from each retina was used as the number of RGCs presented in each eye. Cell counts were performed in a masked manner.

### 2.9. Statistical Analysis

One-way ANOVA followed by a Tukey post hoc test was performed to analyze intragroup differences. The unpaired Student’s t-test was used for the comparison of differences between the two groups. The correlation between RGC counts and IOP exposure was assessed by Pearson’s correlation coefficient. Data are presented as means ± SEM, and *p* < 0.05 was considered statistically significant. 

## 3. Results

### 3.1. Anterior Segment Morphology in egl1 Mice

**Figure 1:**
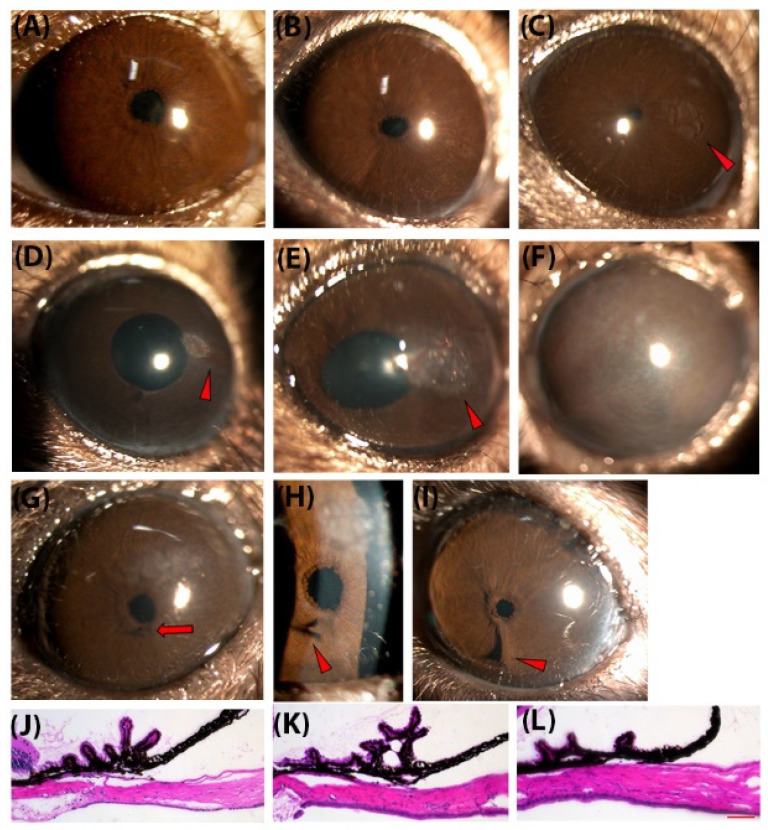
Anterior segment morphology in *egl1* mice. (**A**–**I**) Representative slit lamp image from C57BL/6J (**A**) and *egl1* mice (**B**–**I**). (**A**) Representative slit lamp image showing clear and avascular cornea and centrally located pupil in uniformly shaped iris. (**B**) Normal cornea from an *egl1* mouse. (**C**–**I**) Anterior segment lesions in *egl1* mice. The *egl1* mice developed a variety of anterior segment lesions (indicated by triangles) such as localized faint opacity in paracentral zone of the cornea (**C**), dense corneal opacity with neovascularization (**D**,**E**), iris thinning (**D**,**E**) and pupil deviation (**E**), diffuse opacity covering the entire cornea (**F**), and iris synechiae (the iris adhesion to the cornea) (**G**–**I**). (**H**) Enlarged view of area indicated by arrow in (**G**). (**J**–**L**) Representative H&E staining images of cross-section of the ciliary body and iridocorneal angle from C57BL/6J (**J**), *egl1* mouse with normal IOP (**K**) and after 4 weeks of IOP elevation (**L**). The iridocorneal angle remained open and ciliary body appeared normal triangular in C57BL/6J and the *egl1* mice with normal IOP. Anterior synechiae (the iris attached in the iridocorneal angle) and ciliary body atrophy were seen in eyes with elevated IOP. Scale bar = 50 µm.

We performed slit lamp examination on C57/BL/6J (Figure 1A) and *egl1* mice (Figure 1B–I) aged 4 weeks to 12 months. The *egl1* mice developed anterior segment abnormalities of various types and severities (Figure 1C–I). Approximately 60% of eyes showed localized faint opacity in the paracentral zone of the cornea (Figure 1C). The onset of the corneal lesion was as early as 4 weeks old and appeared independent of mechanical stimulation. Some corneas of aged *egl1* mice remained clear, despite repeated IOP measurements (Figure 1B), similar to those of C57BL/6J wildtype animals (Figure 1A). The corneal lesion progressed to dense corneal opacity with neovascularization in some animals as they aged (Figure 1D,E). In a few animals aged 12 months, we observed the diffuse opacity covering the entire cornea (Figure 1F).

The *egl1* mice also developed iris defects including anterior synechiae (Figure 1G–I), atrophy (Figure 1D), and pupil deviation (Figure 1E). We further examined the anterior segment histology of 8-week-old young adult mice. In the *egl1* mice with normal IOP, the iridocorneal angle remained open, and the ciliary body appeared normal (Figure 1K) compared to C57BL/6J wildtype mice (Figure 1J). In eyes with elevated IOP (Figure 1L), there were anterior synechiae and ciliary body atrophy. Despite the ocular abnormalities, *egl1* animals appeared similar in body size and coat color compared to C57BL/6J mice.

### 3.2. IOP Elevation in egl1 Mice

**Figure 2:**
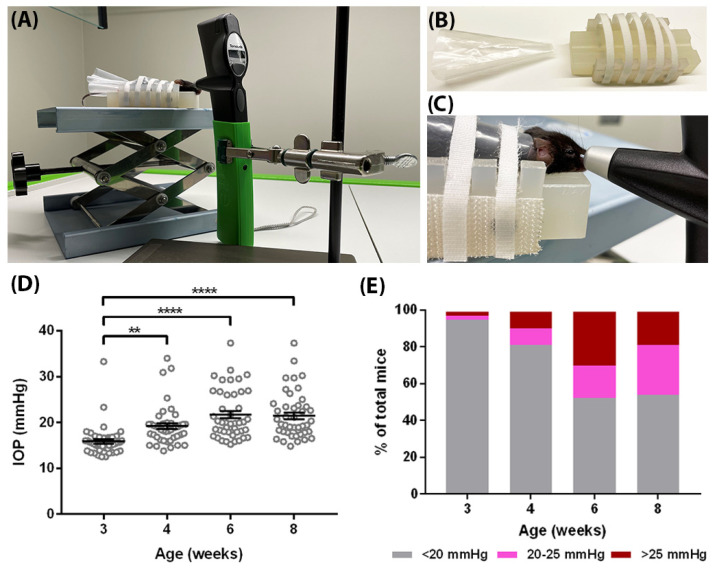
IOP elevation in *egl1* mice. (**A**) Conscious IOP measurement setup. (**B**) Plastic cone and mouse restrainer for IOP measurement. (**C**) Close view of conscious IOP measurement. (**D**) The IOP was monitored in *egl1* mice longitudinally. Significant IOP elevation was detected at 4 weeks of age and remained at all ages examined. Data are presented as means ± SEM (*n* = 44). **: *p* < 0.01, ****: *p* < 0.001. (**E**) The IOP values from each time point were plotted by three ranges, <20 mmHg, 20–25 mmHg, and >25 mmHg. The IOP values demonstrated a wide range in mice of the same age.

The *egl1* mice were maintained on the C57BL/6J background; thus, C57BL/6J mice were used as control animals in this analysis. We monitored the conscious IOP longitudinally in a cohort of 22 *egl1* mice and 6 C57BL/6J mice using the TonoLab tonometer (Figure 2A–C). At 3 weeks of age, *egl1* mice exhibited an IOP of 15.9 ± 0.5 mmHg (mean ± SEM; *n* = 44) which was similar to age-matched C57BL/6J control mice (14.0 ± 1.3 mmHg; mean ± SEM; *n* = 12). A significant elevation of IOP was observed in *egl1* mice as early as 4 weeks old and remain elevated at all ages examined. The IOP values were 19.2 ± 0.6, 21.7 ± 0.8, and 21.4 ± 0.8 mmHg (mean ± SEM; *n* = 44) at 4, 6, and 8 weeks of age, respectively, as shown in Figure 2D. The IOP values remained unchanged in C57BL/6J mice, which were 15.1 ± 0.8, 14.8 ± 1.0, and 15.7 ± 0.9 mmHg (mean ± SEM; *n* = 12) at 4, 6 and 8 weeks of age, respectively. 

The IOP elevation in *egl1* mice was variable and asymmetric. By age 8 weeks, 55% of mice examined exhibited IOPs under 20 mmHg (Figure 2E). The elevated IOP values also demonstrated a wide range in mice of the same age. Some mice had IOPs greater than 35 mmHg at all time points examined. Furthermore, individual animals showed dramatic asymmetry in IOPs, which differed by more than 15 mmHg. 

### 3.3. Aqueous Humor Outflow Facility in egl1 Mice

**Figure 3:**
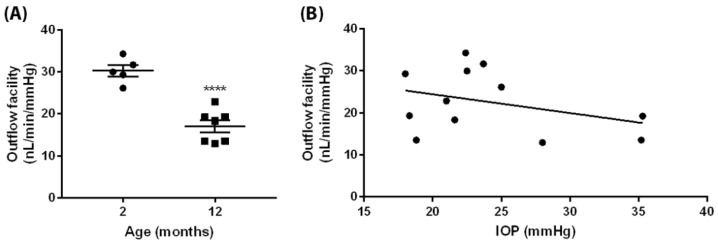
Total aqueous humor outflow facility in young and aged *egl1* animals. (**A**) The aged animals demonstrated significantly lower outflow facility compared to the young animals. Data are presented as means ± SEM (*n* = 5 and 7 for 2- and 12-months old animals, respectively). ****: *p* < 0.001. (**B**) Individual outflow facility was plotted against IOP measurement. The outflow facility and IOP measurement show a trend of negative correlation, but this did not achieve statistical significance.

The AH circulation plays a key role in IOP regulation. We measured total aqueous humor outflow facility in young (2 months) and aged (1 year) *egl1* mice. The outflow facility in young animals was 30.25 ± 1.34 nL/min/mmHg (mean ± SEM; *n* = 5); The aged animals demonstrated significantly reduced outflow facility (17.07 ± 1.44 nL/min/mmHg, mean ± SEM; *n* = 7, *p* < 0.001), indicating a significantly higher outflow resistance in the aged animals (Figure 3A). The outflow facility and IOP measurement showed a trend of negative correlation, but this did not achieve statistical significance (Figure 3B).

### 3.4. RGC Death in egl1 Mice

**Figure 4:**
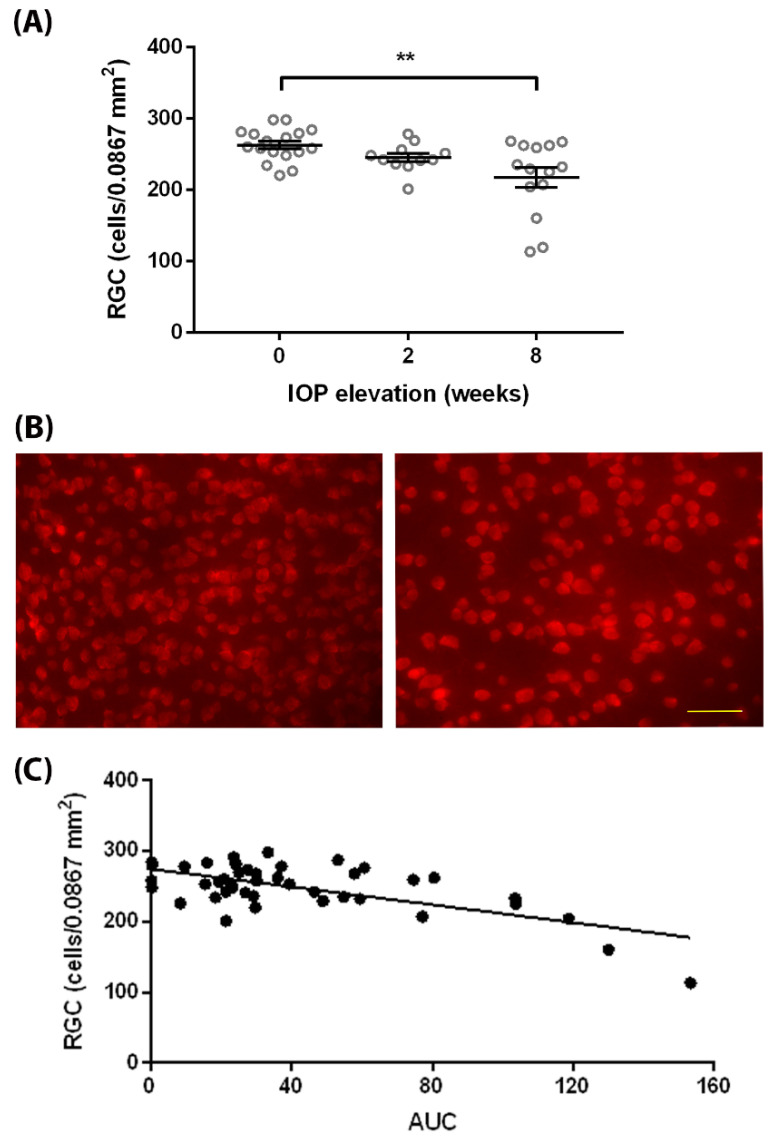
RGC degeneration in *egl1* mice. (**A**) Quantification of RGCs was performed before and at 2 and 8 weeks after IOP elevation. Significant RGC loss was observed after 8 weeks of IOP elevation. Data are presented as means ± SEM (*n* = 11–17). **: *p* < 0.01. (**B**) Representative images show RBPMS (red) immunolabeled retinal whole mounts from mice before (**left**) and 8 weeks after (**right**) IOP elevation. The density of RGCs, demonstrated by RBPMS positive cells, decreased after 8 weeks of IOP elevation. Scale bar = 50 µm. (**C**) Individual RGC count was plotted against IOP exposure described as AUC. There was a moderate negative correlation between RGC counts and IOP exposure. R square: 0.43, *p* < 0.001.

The degeneration of RGCs is a key feature of glaucoma. We quantified the RGC loss in response to IOP elevation in *egl1* mice to assess the glaucomatous neurodegeneration. The *egl1* mice with normal IOPs showed similar numbers of RGCs (263 ± 6/0.0867 mm^2^; mean ± SEM; *n* = 17) in the retina, compared to C57BL/6J control mice (270 ± 8/0.0867 mm^2^; mean ± SEM; *n* = 10). As expected, prolonged exposure to high IOP induced RGC loss in *egl1* mice. RGC numbers (217 ± 14/0.0867 mm^2^; mean ± SEM; *n* = 14) were significantly decreased in mice with 8 weeks of IOP elevation (Figure 4A,B).

To determine the correlation between RGC death and IOP elevation, we further plotted the RGC numbers against IOP exposure calculated as the area under the IOP–time curve. The RGC counts showed a moderate negative correlation (R square: 0.43, *p* < 0.001) with IOP exposure (Figure 4C).

### 3.5. RGC Functional Loss in egl1 Mice

**Figure 5:**
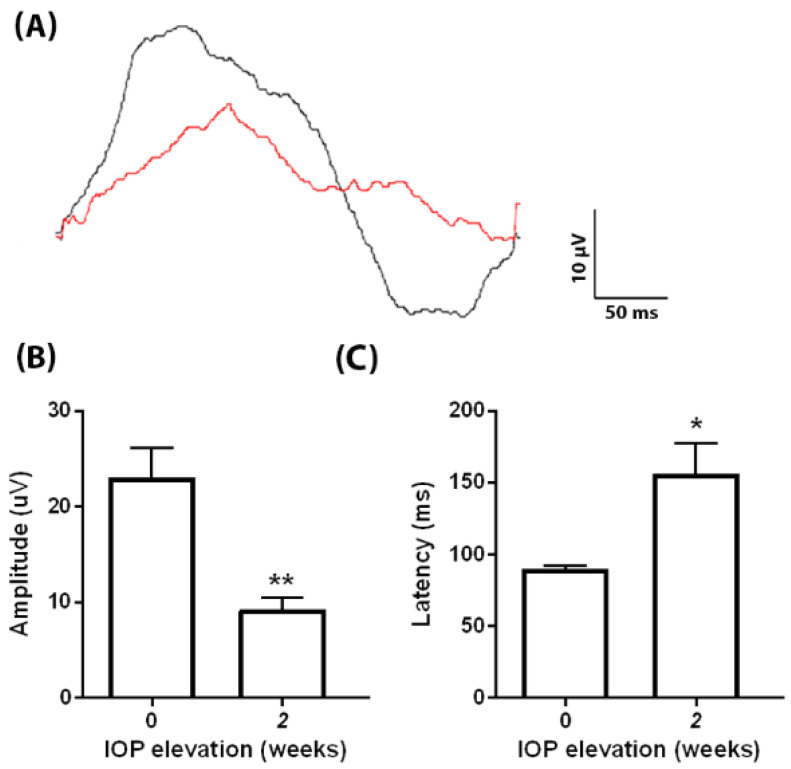
PERG reduction in *egl1* mice. (**A**) Representative PERG waveforms recorded from *egl1* mice before (black) and 2 weeks after (red) IOP elevation. IOP elevation decreased and delayed the PERG response. (**B**,**C**) Bar graphs show decreased PERG responses (**B**) and increased PERG latencies (**C**) after 2 weeks of IOP elevation. Data are presented as means ± SEM (*n* = 5–6) *: *p* < 0.05, **: *p* < 0.01.

To further examine glaucomatous neurodegeneration in *egl1* animals, we performed PERG recording in mice with normal and elevated IOP. The *egl1* animals demonstrated a normal PERG waveform with a positive peak elicited at 89.6 ± 2.4 ms, followed by a negative trough (Figure 5A). The PERG amplitude in the *egl1* mice with normal IOP was 23.0 ± 3.2 µV (mean ± SEM; *n* = 5). After 2 weeks of IOP elevation, there was a significant decrease in PERG amplitude (9.2 ± 1.3 µV) (Figure 5B) as well as an increase in latency (155.7 ± 21.6 ms, mean ± SEM; *n* = 6), which indicates damaged RGC function (Figure 5C).

### 3.6. Glaucomatous ONH Changes in egl1 Mice

**Figure 6:**
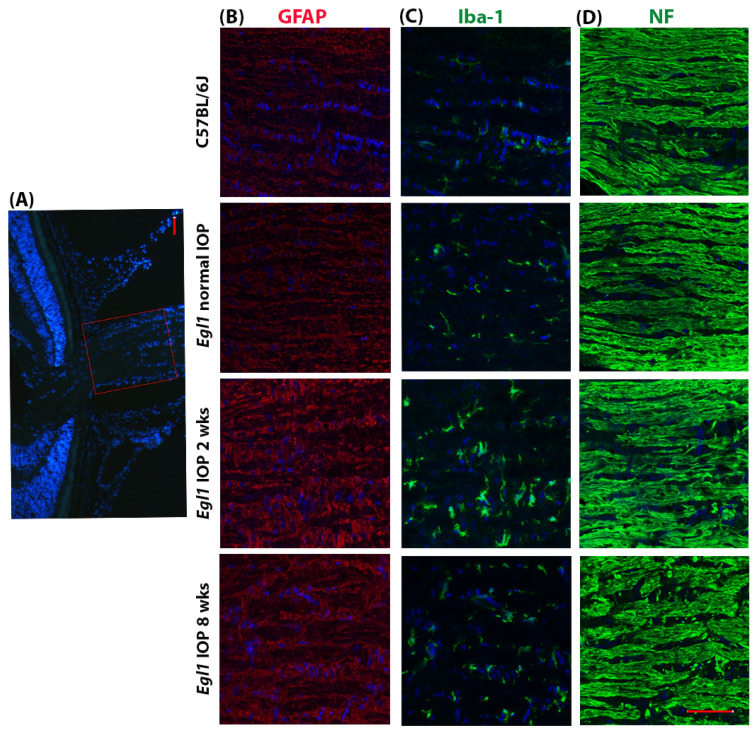
Glaucomatous ONH changes in *egl1* mice. Representative immunofluorescence staining images show increased expression of GFAP and Iba-1 and disorganized NF in the *egl1* mice. Images were taken in the rectangular boxed area in (**A**). (**B**–**D**) Columns show GFAP (red), Iba-1 (green), and NF (green) staining in C57BL/6J wildtype and *egl1* mice with various IOP levels. Nuclei were labeled with DAPI (blue). Scale bars = 50 μm.

Elevated IOP induces glial activation in the ONH and optic nerve axonal degeneration, hallmarks of glaucomatous optic neuropathy. To evaluate these phenotypes in *egl1* mice, we collected eyes from C57BL/6J and *egl1* mice before and 2 and 8 weeks after IOP elevation and performed immunofluorescence staining using specific antibodies against glial fibrillary acidic protein (GFAP), ionized calcium-binding adaptor molecule 1 (Iba-1), and neurofilament (NF) (Figure 6). Eyes of C57BL/6J and *egl1* mice with normal pressure exhibited similar expression levels and patterns of GFAP, Iba-1, and NF. Elevated IOP increased expression levels of GFAP and Iba-1, which peaked at 2 weeks, indicating astrocyte activation and microglia infiltration in the ONH in response to glaucomatous insult. Quantification of GFAP and Iba-1 fluorescence intensity showed a trend of increase at 2 weeks after IOP elevation without statistical significance (*n* = 3–6). The pattern of neurofilaments became disorganized after 2 weeks of IOP elevation. Eyes with extended pressure elevation further exhibited disruption of neurofilaments, suggesting optic nerve axonal damage in the *egl1* mice.

## 4. Discussion

The present study characterized the glaucoma phenotype in a mouse strain carrying a missense mutation of *Pitx2* named the *egl1* mutation. The *egl1* mutant mice developed glaucoma with a significant variation in the age of onset and phenotypic severity within and between animals. The mice with ocular hypertension demonstrated glaucomatous retinopathy and optic nerve neuropathy.

As a powerful tool, mouse models carrying the *Pitx2* mutation or deletion have been developed to investigate the functions of *PITX2* and the pathogenesis of glaucoma. The *egl1* mice have a chemically induced *Pitx2* mutation and develop early-onset glaucoma. The mouse strain is homozygous for the *egl1* mutation, which was mapped to chromosome 3. High-throughput sequencing further identified the mutation as a single G to T transversion in *Pitx2* exon 2, changing amino acid 115 from arginine to leucine [26,27]. In humans, *PITX2* has been identified as a glaucoma-causing gene. Mutations in *PITX2* are associated with Axenfeld–Rieger syndrome, which involves ocular malformations leading to congenital and childhood glaucoma in more than 50% of the affected patients [40]. Similarly, we found that the *egl1* mouse strain developed anterior segment abnormalities, and by the age of 6 weeks, approximately 50% of the animals developed early-onset glaucoma despite having an identical genotype and genetic background. Gender difference in the risk for glaucoma and glaucoma blindness has been well documented [41,42]. In the *egl1* mice, ocular hypertension developed in male and female mice at a similar percentage. Larger-scale studies are needed to reveal potential sex-associated differences in glaucoma development and severity in *egl1* animals. The IOP elevation in *egl1* animals exhibited significant variation and asymmetry. The onset of ocular hypertension was as early as 3 weeks old, and IOP was elevated to a maximum of 20 to 37 mmHg. Individual animals exhibited significant ocular hypertension in one eye and normal IOP in another. The variation and asymmetry in the development of ocular hypertension may require larger animal cohorts in the research design to ensure a sufficient sample size for statistical analysis. On the other hand, the wide range of IOP values offers a spectrum of disease severity and eyes with normal tension, providing an ideal strain-matched control.

Through our longitudinal IOP measurements, we also found that the IOP elevation in the *egl1* mice demonstrated two patterns: sustained mild elevation until the age of 4 months or high elevation followed by normal pressure. Ciliary body atrophy has been described in the *Pitx2*^+/−^ mice as well as in human chronic cases when left untreated [25]. Similarly, the histologic examination showed ciliary body atrophy in the *egl1* mice with prolonged ocular hypertension. The reduction of IOP following a high pressure elevation in the *egl1* mice is likely due to ciliary body atrophy. Aqueous humor is produced by the epithelium of the ciliary body and drained primarily through the conventional outflow pathway consisting of the trabecular meshwork (TM), Schlemm’s canal, the scleral collector channels, and aqueous veins, sequentially. It has been recognized that increased aqueous humor outflow resistance is associated with elevated IOP in glaucoma [43,44]. In addition, studies in humans and monkeys have shown that aqueous outflow facility (the reciprocal of aqueous humor outflow resistance) naturally declines with aging, even in healthy eyes [45,46]. Thus, we measured the AH outflow facility in young and aged animals and assessed the correlation between IOP and AH outflow facility. There was a negative trend of correlation between IOP and AH outflow facility, suggesting a role played by declined outflow facility in IOP elevation in the *egl1* mice. However, we did not detect a statistically significant correlation between IOP and facility.

Pattern ERG is a well-accepted standard for assessing RGC function. It allows non-invasive and longitudinal evaluation of RGC function. It detects RGC functional loss prior to significant morphological damage [47]. Using PERG, we detected RGC functional loss at 2 weeks after IOP elevation when a significant RGC death was undetectable. The results further demonstrate the glaucomatous phenotype in *egl1* animals and support the use of PERG as a sensitive tool to monitor the disease progression in the *egl1* mouse strain.

The quantification of RGCs showed a mild correlation between RGC loss and IOP exposure. In addition, outer retinal dysfunction has been reported in *egl1* mice. At 3 months of age, there is a decreased rod b-wave and a lower cone response, which indicates a possible outer retinal degeneration [27]. A separate study also showed that the deletion of the *Pitx2* gene disrupts the development of the retinal pigment epithelium [23]. It is also possible that outer retinal degeneration may in turn affect RGC health within the inner retina and contribute to the decrease of RGC numbers.

Recent studies have shown that glia play an important role in the pathogenesis of glaucoma [48,49,50,51]. Astrocytes, the major glial cell population in the ONH, are considered the mediators of axonal injury in glaucoma. They respond to mechanical compression due to IOP elevation and undergo a number of cellular changes, such as upregulation of GFAP cytoskeleton and neurotrophic or neuroinflammatory factors [49,52]. Similarly, microglia in the ONH become activated and redistributed, express cytokines and other secreted factors, and exert neuroprotective or neurotoxic effects in glaucomatous eyes [51]. In the *egl1* animals, we found upregulated GFAP, and Iba-1 expression also peaked at an early stage of the disease, suggesting the activation of astrocytes and infiltration of microglia following glaucomatous insults. This further validates the glaucoma phenotype in the mutant animals. The results also support the use of *egl1* mice as an in vivo model to investigate mechanisms of glial activation in glaucoma.

In summary, this study demonstrates that the *egl1* mouse strain exhibits key features of early-onset glaucoma and provides an important tool to study glaucomatous neurodegeneration. Additional studies are needed to further characterize glaucomatous neuropathy in *egl1* animals.
